# A method for evaluating the toxicity interaction of binary mixtures

**DOI:** 10.1016/j.mex.2020.101029

**Published:** 2020-08-13

**Authors:** Qiu-yang Wei

**Affiliations:** a Chongqing Academy of Chinese Materia Medica, China; b Southwest University, China

**Keywords:** Bioassay, Co-toxicity coefficient (CTC), Synergism

## Abstract

Improving the formulation of biological insecticides for greater efficiency and competitiveness is of particular importance with respect to the successful application of these agents in the field. In this regard, mixing different agents is known to be an effective strategy for enhancing practical pest control. However, traditional chemical-based control strategies have significant limitations that compromise control effectiveness, as effects tend to become increasingly dose-dependent over time. To overcome such limitations, and thereby ensure the continuous effective control of insect pests, we herein assessed the efficacy of binary mixtures of biological agents, with the aim of establishing an optimal ratio. The optimized mixture showed a significantly higher insecticidal effect, whereby biological pest control was considerably enhanced.

• We combined *Beauveria bassiana* spores with azadirachtin, a chemical pesticide of botanical origin, and by assessing the efficacy of these two elements combined in different ratios, obtained an optimal formula. Additionally, we evaluated the compatibility between the two elements, and then assessed potential synergistic effects, as determined by the co-toxicity coefficient.
• This protocol is dependent on the results of bioassays. It is not only suitable for combining fungal and chemical agents for the purposes of biocontrol but is also applicable to a variety of biological pesticides.
• The evaluated binary mixture showed enhanced effective insect control. This approach will contribute to reducing applied dosages, thereby promoting efficient pest control and reducing application costs.

## Graphical abstract

**Figure:**
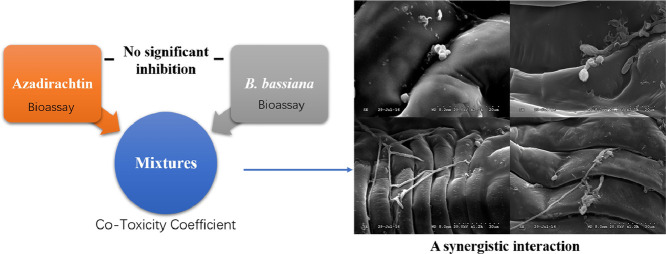

**Table:** Specifications Table

Subject Area	Agricultural and Biological Sciences
More specific subject area	*Biological pest control*
Method name	A dual agent mixing method using microbial and chemical agents
Name and reference of original method	Analysis of mixtures containing two insecticides based on co-toxicity coefficient determinations Our method is an enhanced version. The improved method is particularly suitable for compounding microorganisms and chemical compounds. Gu Z-Y, Chen M-L; Xu X-L, Xu D-J, Xu G-C. (2010). Deviation of co-toxicity coefficient in evaluation of joint action of pesticides against pests and its correction. Jiangsu J. Agr. Sci 26(6), 1238-1246. DOI: CNKI: SUN: JSNB.0.2010-06-022 Perumalsamy H, Kim J-R, Kim S-L, Kwon H-W, Ahn Y-J. (2012). Enhanced toxicity of binary mixtures of larvicidal constituents from *Asarum heterotropoides* root to *Culex pipiens pallens* (Diptera: Culicidae). J Med Entomol 49(1), 107-111. DOI: 10.1603/ME11092 The two methodologies are based on co-toxicity coefficient evaluation.
Resource availability	

## Background

The use of microbial agents for insect pest control has received increasing attention in recent years, as a consequence of increased public awareness regarding residue, resistance, and resurgence (3R) problems, particularly with respect to the resistance of pests to chemical insecticides, and the potential of microbial agents to provide effective environmentally friendly pest control [1]. Accordingly, a variety of biological control organisms are now commercially applied in the field, including: predatory arthropods (or mites), parasitic insects, and insect-pathogenic microorganisms, among which, entomogenous fungi often have the advantage of ease of application and low cost. Therefore, the use of microbial agents has become a preferential strategy for agricultural pest management. As the beneficial effects of microbial insecticidal agents generally develop slowly, mixtures of such microbial agents with botanical insecticides are predicted to be a more effective solution [2]. In this regard, the nymphs of whiteflies (*Trialeurodes vaporariorum*), which do not crawl or move, are particularly susceptible upon exposure to pathogenic microbes, whereas in contrast, once they reach the adult stage, whiteflies can easily avoid microbial infestation by flying. Therefore, entomopathogenic fungi are most effective for the control of whitefly at the nymphal stage.

## Method details

### Reagents and equipment

Azadirachtin EC 0.5% (Yunnan Guangming Neem Industry Development Co., Ltd)
*Beauveria bassiana* spores (isolate Bb252)
Tween-80 (Macklin Co., Ltd)
Potato dextrose agar (medium) (NEST Biotechnology Co., Ltd)
Beaker
Graduated cylinder
Volumetric flask
Petri dish
Absorbent cotton
Artificial climate box (RDN-260A; Ningbo Dongnan)
Biochemical incubator (RSZ-10; Ningbo Dongnan)
Microscope (Primotech; ZEISS)
Haemocytometer

### Preparation

#### Insects and plants

*Trialeurodes vaporariorum* individuals were collected from the leaves of tobacco growing in the Fengjie area of Chongqing, China. Identification of the species was based on cytochrome *c* oxidase (COI) gene barcoding, which confirmed *T. vaporariorum* (GenBank submission: KX024590) identity. Whiteflies were bred for more than 30 generations in the laboratory on tobacco cv. ‘Yunyan 87’, cultured at 26 °C ± 1 °C and RH = 75% ± 7% under a 14:10 h light/dark cycle. Tobacco plants were used in trials when they had reached a height of 25 cm. Prior to the beginning of the trials, 50 adult whiteflies were released onto a tobacco seedling and allowed to lay eggs for 24 h, after which the adults were removed, and trials commenced when the eggs had hatched and the nymphs had reached third-instar stage.

#### Fungus and azadirachtin

*Beauveria bassiana* (isolate Bb252), which has been isolated from a range of herbivorous insect host species (including *Tetranychus urticae, Chilo suppressalis, Sitophilus granarius*, and *Myzus persicae*), was used in these experiments and stored at the Biotechnology Centre of Southwest University at −80 °C. *B. bassiana* was initially isolated from *C. suppressalis* feeding on maize in the Yongchuan District of Chongqing, China, and was separated into single spores. For all trials, Bb252 was grown in the dark on potato dextrose agar (PDA) medium (pH = 6.5–6.7) at 28 °C ± 1 °C. We applied 0.1% Tween-80 to emulsify the blastospores and determined the concentration using a haemocytometer.

The plant-based pesticide used in the bioassay contained 0.5% azadirachtin EC (Yunnan Guangming Neem Industry Development Co., Ltd).

### Experimental procedure

#### Bioassay of *B. bassiana* and azadirachtin

A normalized dose-response method was used for evaluating the efficacy of *B. bassiana* Bb252 and azadirachtin. Determination of fungal pathogenicity was based on a gradient comprising five concentrations of spores: 1 × 10^4^, 1 × 10^5^, 1 × 10^6^, 1 × 10^7^, and 1 × 10^8^ conidia/mL. Similarly, the lethality (median lethal concentration, i.e., LC_50_) of azadirachtin was evaluated over a range of concentrations (0.5, 1.0, 5.0, 50, and 500 mg/L), with sterile water being used as the control treatment. Separate batches of 30 third-instar nymphs were immersed three times in the treatment solutions, each for 10 s. Thereafter, the treated nymphs were placed in culture dishes (9 cm diameter) and stored at 27 °C ± 1 °C and RH = 75% ± 7% under a 14:10 h light/dark cycle. Nymph mortality was assessed after 7 days.

#### Effect of azadirachtin on the growth rate of *B. bassiana*

An experiment was performed to assess the effects of the aforementioned concentrations of azadirachtin on the growth of *B. bassiana*. Petri dishes (10 mL) containing PDA medium supplemented with azadirachtin at each of the five concentrations were inoculated with a plug of fungal mycelium, which was cut using a 0.5-cm-diameter) puncher, with five replicate plates being prepared for each insecticide concentration. The dishes were then placed in a dark biochemical incubator (28 °C ± 1 °C, RH = 75% ± 7%). After 9 days, the diameter of the fungal colonies was measured, and inhibition rates were calculated according to the following formula: inhibition rate (%) = (control – test)/control × 100. Furthermore, spores on the medium were collected and used to prepare spore suspensions (1 × 10^4^ conidia/mL). After a 3-day period of culture (28 °C ± 1 °C, RH = 75% ± 7%), a drop of the conidial suspension was placed in a haemocytometer and the percentage rate of conidial germination was calculated using the following formula: spore germination rate (%) = (number of germinated spores/number of observed spores) × 100 [3].

#### Toxicity interaction of binary mixtures of azadirachtin and *B. bassiana* against whiteflies

All toxicity interaction studies were based on LC_50_ values for third-instar nymphs exposed to a 7-day treatment. As binary combinations, azadirachtin and *B. bassiana* were mixed in the following ratios: 9:1, 4:1, 1:1, 1:4, and 1:9 [3]. The prepared stock solution was serially diluted 10-fold to give a concentration gradient of 1 ×, 10 ×, 100 ×, 1000 ×, and 10000 ×, and mixtures at these five concentrations were applied in dose-mortality testing, with each binary combination being assessed in triplicate. All samples were incubated at 26 °C ± 1 °C and assessed after 7 days. A linear fitting equation was obtained based on assessments of multiple dilutions, and using this, LC_50_ values were estimated [4]. Values for the co-toxicity coefficient (CTC), which were based on the 7-day LC_50_ value for each binary mixture, were used to determine interaction efficiency. A CTC value higher than 120 indicated marked synergism, whereas a value between 80 and 120 was considered cumulative, and a value of less than 80 was taken to be indicative of antagonism [5,6]. As each mixture (M) was formulated with parts A and B, the CTC value for M was calculated as follows:

Toxicity index (TI) of A = 100;
TI of B = LC_50_ of A/ LC_50_ of B × 100;
Actual TI of M = LC_50_ of A/ LC_50_ of M × 100;
Theoretical TI of M = TI of A × % (W) of A in M + TI of B × % (W) of B in M;
CTC = Actual TI of M/Theoretical TI of M × 100.
Note: (W) represents the agent ratio in mixture.

### Statistical analysis

Average of insect mortality was calculated by linear regression fitting and all the values were corrected with respect to the control using Abbott's formula (percentage corrected control = (% alive in the control – % alive in the treatment/% alive in the treatment) × 100) [7,8].

### Method validation

#### Comparison with pesticide control effect

By fitting the data to the virulence equation, the LC_50_ for the mixed formulations was found to range from 0.22 to 50.63 mg/L (Table 1), whereas CTC values ranged between 41.03 and 294.23. Maximum values for both indices (CTC: 294.23 and LC_50_: 0.22 mg/L) were obtained for the binary mixture prepared using an azadirachtin to *B. bassiana* mixing ratio of 1:4, thereby indicating a synergistic effect. However, the LC_50_ for the azadirachtin treatment alone was 5.76 mg/L (*R*-sqr=0.998, *Chi*-sqr = 1.51349). Pesticide used alone required higher doses than mixed formulas to obtain an optimal response.

**Table 1:** The virulence of mixtures of different ratios of azadirachtin and *Beauveria bassiana* against whiteflies and the corresponding co-toxicity coefficients.

Mixture ratios (A: B)	Regression equation	*r^2^*	LC_50_ (mg/L)	Co-toxicity coefficient (CTC)

9:1	*y* = −0.35*x* + 50.5	0.94	7.42	54.41
4:1	*y* = −0.12*x* + 51.3	0.99	50.63	86.60
1:1	*y* = −3.77*x* + 54.1	0.96	3.32	104.18
1:4	*y* = −3.92*x* + 50.6	0.93	0.22	294.23
1:9	*y* = −3.90*x* + 61.1	0.94	2.66	41.03

Note: A: azadirachtin; B: *Beauveria bassiana.*

#### Observation of fungal infection cycle

**Fig. 1:**
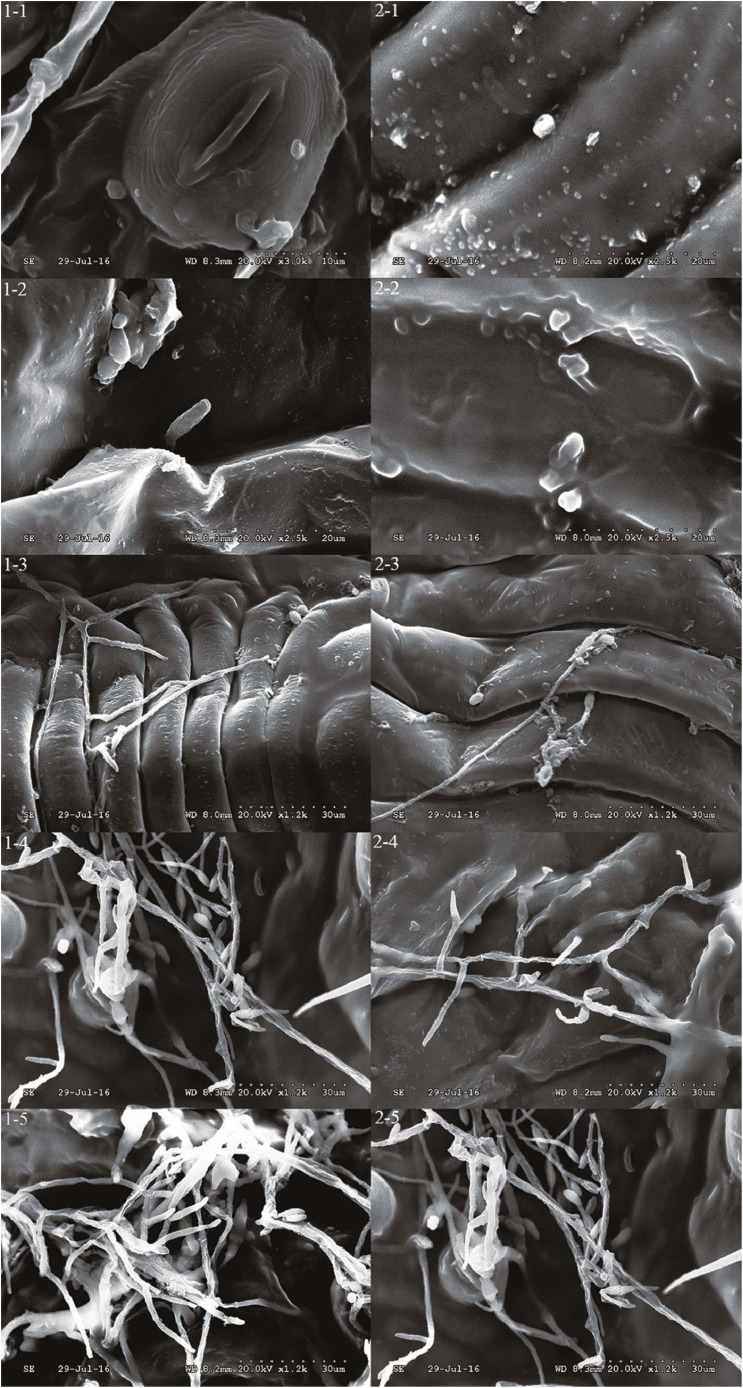
Proliferation of *Beauveria bassiana* conidia on whiteflies. 1. *B. bassiana* conidia only. 2. A mixture of azadirachtin and *B. bassiana* at a ratio of 1:4. 1–1 and 2–1: conidia on the cuticle of whitefly nymphs (24 h); 1–2 and 2–2: penetration of germinated conidia (48 h); 1–3 and 2–3: growth of hyphae (72 h); 1–4 and 2–4: hyphae colonising the entire host insect (96 h); 1–5 and 2–5: development of fungal hyphae into a dense network (120 h).

To assess the effects of the plant-derived compound azadirachtin on fungal spore germination, scanning electron microscopy (SEM, Hitachi S-3000N10100) was used to observe the conidia that had adhered to the whitefly nymphs, which became infected following spore germination. For tissue fixation, all nymphs were fixed with 2.5% glutaraldehyde. Prior to observation, the samples were dried by vacuum freeze-drying (SJIA-10N-60A; Ningbo Shuangjia). Observations revealed that spores which were mixed in the optimal 1:4 ratio had a significant infection rate. The complete spore infection cycle could be observed more clearly in the mixed formula samples (Fig. 1). These results indicate that the combination of azadirachtin and *B. bassiana* have great potential for application in insect pest control.

## Declaration of Competing Interest

The authors declare that they have no known competing financial interests or personal relationships that could have influenced the work reported in this paper.

## Appendix Supplementary materials

Image, application 1
